# DIssociation of T lymphocyte subpopulations in patients with juvenile idiopathic arthritis

**DOI:** 10.1186/1546-0096-12-S1-P210

**Published:** 2014-09-17

**Authors:** Bulegenova Minira, Akhenbekova Aida

**Affiliations:** 1 Rheumatology, Scientific center of pediatrics and children surgery, Almaty, Kazakhstan

## Introduction

Introduction and objective: it is well known fact that the key point in the development of an autoimmune response in rheumatoid inflammation is the dissociation between subpopulations of T lymphocytes.

## Objectives

The aim of our study was to analyze the quantitative changes in the spectrum of T-lymphocytes and the activity of the pathological process in children with juvenile idiopathic arthritis ( JIA ).

## Methods

Materials and Methods: the main subpopulations of T - lymphocytes in peripheral blood were determined by laser flow cytometer - FacsCalibur using the program Cell-Quest. The study was conducted in patients with different stages of JIA. A following panel of antibodies: CD45/CD14, IgG1/IgG2,; CD3/CD19, CD4/CD8, CD3/HLA-DR, CD16/56, CD71 , CD95/CD54 , CD38 was used to identify lymphocyte populations.

## Results

Results of our study revealed the elevated levels of lymphocytes expressing CD3 + CD19 markers - 27,3 ± 3,4% ( compared with the reference parameters - 9.5 ± 1.1%). Besides, decreasing of CD3+19-T-lymphocytes (51,6 ± 2,4% compared to healthy 76,2 ± 1,5%), was in direct correlation with the high activity of the process ( P <0.05). Moreover, it was necessary to define two groups of results:

1 - a significant increase in T-helper cells ( CD4 + CD8-) to 44,9 ± 4,2% ( control group -34.7 ± 2.1%) while the number of CD8 + Tcytotoxic cells was within normal parameters . These results indicate the predominant contribution of 2and 3 types hypersensitivity, which are cha-racterized with the production of autoantibodies during the pathology process.

2 - preservation of T -helper population within the reference values while the content of T CD8 + effectors was increased that indicates the cell type of hypersensitivity. Growth of CD8 + T cells correlated with the activity of the process, while remaining normal in oligoarthritis with low laboratory activity (ESR, CRP). Deterioration of articular changes followed by increased levels of CD95+T-lymphocytes (12,8 ± 1,9% when a rate of healthy is 3,2 ± 0,6%). In our opinion, direct correlation between the CD95+T lymphocytes and CD8+Tcytotoxic cells indicated the dependence between proliferation, cytotoxicity and apoptosis . The level of activated CD3 + HLA-DR+ T cells was significantly increased in JIA up to 9,7 ± 1,5% ( com-pared with healthy children - 4 , 1 ± 0,5%). In one patient with systemic JIA (stage of severe rheumatoid inflammation) the level of activated CD3 + HLA-DR + T lymphocytes increased dramatically up to 38.7 %. It is necessary to point that our results did not reveal the growth of the serum immunoglobulins.

## Conclusion

Conclusion: dissosiation of T-lymphocyte subpopulations in children with JIA correlated with clinical activity of the disease. Screening of T lymphocytes populations is promising for a personified therapy selection in patients with JIA **.**

## Disclosure of interest

None declared.

